# Cervical cancer screening program in Thimphu, Bhutan: population coverage and characteristics associated with screening attendance

**DOI:** 10.1186/s12905-014-0147-0

**Published:** 2014-11-30

**Authors:** Iacopo Baussano, Ugyen Tshomo, Gary M Clifford, Vanessa Tenet, Tshokey Tshokey, Silvia Franceschi

**Affiliations:** International Agency for Research on Cancer, 150 cours Albert Thomas, 69372 Lyon Cedex 08, France; Department of Obstetrics & Gynaecology, Jigme Dorji Wangchuck National Referral Hospital, Thimphu, Bhutan; Department of Laboratory Services, Jigme Dorji Wangchuck National Referral Hospital, Thimphu, Bhutan

**Keywords:** Screening, Cervical cancer, Bhutan

## Abstract

**Background:**

Bhutan has been engaged in good-quality cytology-based cervical screening since 2000 and has vaccinated >90% girls against human papillomavirus (HPV) since 2010. We explored the characteristics associated with lack of previous screening and screening coverage in women age ≥25 years.

**Methods:**

Women were invited at home or during their attendance at 2 outpatient clinics, in the capital, Thimphu, and nearby Lungthenphu. Age-adjusted odds ratios for lack of previous screening by selected characteristics were computed among 1,620 participating women. In Thimphu an invitation registry allowed to estimate screening history not only among participating women but also among additional 500 women who did not accept to join our study.

**Results:**

Among women who had a Pap smear, lack of previous screening was associated with age <35 or ≥45 years. It was also associated with some occupations; being single, or widowed/separated; and presence of HPV infection. Multiparity and use of contraceptive methods were associated with having been screened. In women invited at home in Thimphu screening history substantially differed by participation. Past screening attendance was 59% among women recruited in the 2 clinics, 53% in women who were invited from home and accepted the invitation, but only 25% in those who refused it. Based on all women recruited from home the estimate of population-based coverage in Thimphu is 34% (95% CI: 31-37).

**Conclusions:**

Transition from an opportunistic screening to an all-reaching population-based screening is yet to be achieved in Bhutan, even in the capital. Better ways to target never-screened women are needed.

## Background

Cervical cancer represents the most common cancer among women in Bhutan, with a world age-standardized incidence rate of 13 cases per 100,000 person years [1]. Cervical cancer incidence is likely to be underestimated on account of the incomplete establishment of the national cancer registry. The age-standardized prevalence of human papillomavirus (HPV) infection in the general female population in Bhutan is 26%, ranging from 33% below age 25 years and 19% at age 45 and above [2]. It is one of the most elevated HPV prevalence among Asian countries [3-9]. It is therefore very appropriate that the country (634,982 inhabitants) has seriously engaged in cervical cancer prevention [10]. Bhutan was the first low/middle-income country to start in 2010 a nationwide school-based program of vaccination against HPV and has achieved vaccination-coverage of 92% among 12 to 18 year old girls [11].

Bhutan has also been on the front line in the secondary prevention of cervical cancer: a national cytology-based screening program, including quality control of Pap smear findings, and systematic recall of screening-positive women for colposcopy and, if necessary, biopsies and treatment, was launched in 2000 [10]. No active call/recall system is in place but a Pap smear is recommended every three years to women aged 20-60 years and it is provided free of cost by trained female health assistants, nurses, and medical doctors in national referral hospitals and district hospitals through maternal and child health clinics. Although Pap smear campaigns are occasionally conducted in rural and high-altitude areas, the majority of screening activity is concentrated in the district of the capital, Thimphu, which hosts approximately one sixth of the Bhutanese population. The only information on the performance of cervical cancer screening in Bhutan is a recent publication that shows poor knowledge of and low participation in cervical cancer screening among female university graduates, mainly below age 25 years [10].

The aims of the present study were to provide an estimate of screening coverage in Thimphu and explore the characteristics associated with lack of previous screening among women age 25 or older. The evaluation of screening was part of a large HPV survey carried out by the Jigme Dorji Wangchuck National Referral Hospital (JDWNRH), Thimphu, in collaboration with the Ministry of Health of Bhutan, and the International Agency for Research on Cancer (IARC) to monitor future impact of HPV vaccination and the transition from cytology to HPV testing in cervical cancer screening program.

## Methods

**Figure 1:**
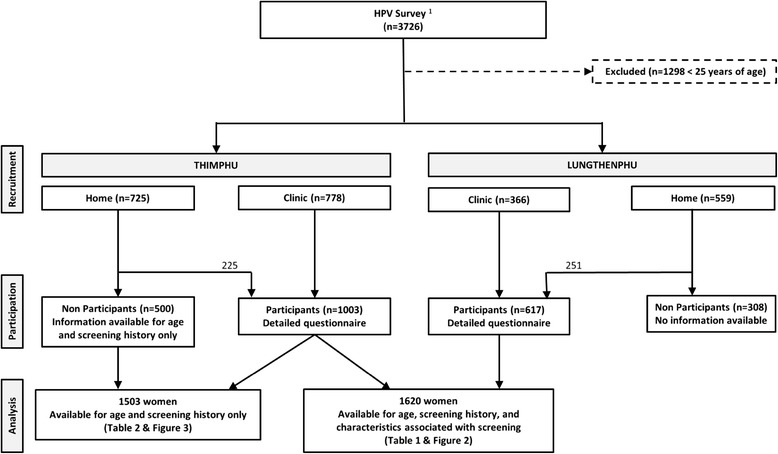
**Diagram of Bhutan screening survey.** ^1^Tshomo et al, 2014.

Between December 2011 and October 2012, 2,505 sexually active women age 18-69 years underwent conventional cytology and HPV testing in JDWNRH, Thimphu, and in the Hospital of Lungthenphu, a satellite town in the district of Thimphu, to provide baseline HPV prevalence prior to HPV vaccination. Study procedures have been described elsewhere [2]. In brief, women in both study areas were recruited in two ways (Figure 1). Residents of pre-defined areas surrounding the two hospitals were visited at home by social workers and invited to join the study (participation: 31% in Thimphu and 45% in Lungthenphu). Women consulting outpatient clinics in the two hospitals for cervical screening (mainly antenatal care or family planning clinics) were invited to join the study (participation ~ 100%). Following signature of an informed consent form, a structured questionnaire including information on screening history and other socio-demographic and lifestyle characteristics was administered to study participants. Testing for 44 HPV types was made using a general primer GP5+/6+ -mediated PCR and reverse-line blot hybridization [12].

To investigate screening participation in recommended age groups [13], the present report focused on 1,620 women aged 25 years or above (median age = 33, inter-quartile range: 28-43) [2]. Among them, 1,003 were seen at the JDWNRH and 617 at the Lungthenphu Hospital. In Thimphu only, an invitation registry including age, marital status, and screening history was kept and allowed the additional evaluation of all 500 women 25 years or above who had been invited at home in Thimphu but did not eventually participate in the study (Figure 1).

Odds ratios (OR) for lack of previous screening and corresponding 95% confidence intervals (CI) were computed by unconditional logistic regression adjusted for 5-year age groups. Additional adjustment for type of recruitment did not materially modify any findings. *χ*^2^ test for trend were computed by considering categories as continuous variables. Effect modification was tested using the log-likelihood ratio test.

The present study had the approval of both the Bhutan Research Ethics Board of Health and the IARC Ethics Committee.

## Results

Overall, 661 (40.8%) women who took part in our study had never been screened before. Table 1 shows the relationship between screening history and selected women’s characteristics among study participants. No significant difference was found between the two study areas. The lowest fraction of never-screened women was found in the age group 35-44 years (28.0%). Lack of previous screening was more frequent in women below age 35 years and those age 45 or above (OR *versus* age 35-44 = 2.3; 95% CI:1.8-3.0; and 1.7; 95% CI: 1.2-2.3, respectively). Compared to housewives, the fraction of never-screened women was larger in shopkeepers/saleswomen (OR = 1.6; 95% CI: 1.1-2.3) and manual workers/farmers (OR = 1.7; 95% CI: 1.2-2.4). Among ethnic groups, a relative lack of previous screening was found in Lhotsampa (OR *vs.* Scharchop = 1.6; 95% CI: 1.2-2.1). ORs for lack of previous screening in single and widowed/separated women *vs.* married women were 4.9 (95% CI: 1.3 - 17.9), and 1.8 (95% CI: 1.2 - 2.6), respectively.

**Table 1:** **Odds ratio and 95% confidence interval for lack of prior cervical cancer screening by selected women’s characteristics**, **1,620 women, Thimphu and Lungthenphu, Bhutan**

**Variable**	**Total**	**N. (%) of women never screened**	**Odds ratio** ^**a**^	**95% confidence interval**
**All women**	1620	661 (40.8)	–	–
**Recruitment area**				
Thimphu	1003	426 (42.5)	Ref. cat.	
Lungthenphu	617	235 (38.1)	0.8	0.7 – 1.0
**Age groups (yrs)**				
25-34	852	407 (47.8)	2.3	1.8 – 3.0
35-44	435	122 (28.0)	Ref. cat.	
≥45	333	132 (39.6)	1.7	1.2 – 2.3
**Occupation**				
Housewife	1051	402 (38.2)	Ref. cat.	
Shopkeeper/saleswoman	161	77 (47.8)	1.6	1.1 – 2.3
Manual worker/Farmer	154	81 (52.6)	1.7	1.2 – 2.4
Clerical staff	110	44 (40.0)	1.0	0.6 – 1.5
Teacher/Health worker/Students	131	48 (36.7)	1.0	0.6 – 1.4
**Ethnic group**				
Scharchop	605	226 (37.4)	Ref. cat.	
Ngalop	396	168 (42.4)	1.3	1.0 – 1.7
Lhotsampa	340	156 (45.9)	1.6	1.2 – 2.1
Other	274	106 (38.7)	1.1	0.8 – 1.5
**Marital status**				
Married	1487	586 (39.4)	Ref. cat.	
Single	15	12 (80.0)	4.9	1.3 – 17.9
Widowed/separated	112	57 (50.9)	1.8	1.2 – 2.6
**Pregnancies**				
0	93	63 (67.7)	1.9	1.2 -3.2
1	267	145 (54.3)	Ref. cat.	
2	442	182 (41.2)	0.7	0.5 – 0.9
≥3	809	263 (32.5)	0.6	0.4 – 0.8
${\chi}_1^2$ for trend			= 9.8, p = 0.002^b^	
**Contraceptive method**				
None	343	194 (56.6)	Ref. cat.	
Hormonal	656	261 (39.8)	0.5	0.3 – 0.6
Other	606	196 (32.3)	0.4	0.3 – 0.5
**Lifetime sexual partners**				
1	1420	568 (40.0)	Ref. cat.	
≥2	174	77 (44.2)	1.4	1.0 – 1.9
**HPV infection**				
Negative	1254	479 (38.2)	Ref. cat.	
Positive	366	182 (49.7)	1.5	1.2 – 1.9

Note: Some figures do not add up to the total due to missing values; ^a^Adjusted for age, as appropriate.

^b^Trend assessed among ever pregnant women only.

The fraction of never-screened women decreased with number of pregnancies (*χ*^2^ for trend among women who had been pregnant = 9.8, p = 0.002) and it was largest among nulligravidae (OR for 0 *vs.*1 child = 1.9; 95% CI: 1.2-3.2). Fewer never-screened women were found among current users of any contraceptive method (OR in users of hormonal contraceptives and other methods *vs.* non users = 0.5, 95% CI: 0.3-0.6; and 0.4; 95% CI: 0.3-0.5, respectively). Finally, lifetime sexual partners (OR for ≥2 *vs* 1 = 1.4; 95% CI: 1.0-1.9) and presence of HPV infection (OR = 1.5; 95% CI: 1.2-1.9) were associated with lack of previous screening (Table 1).

**Figure 2:**
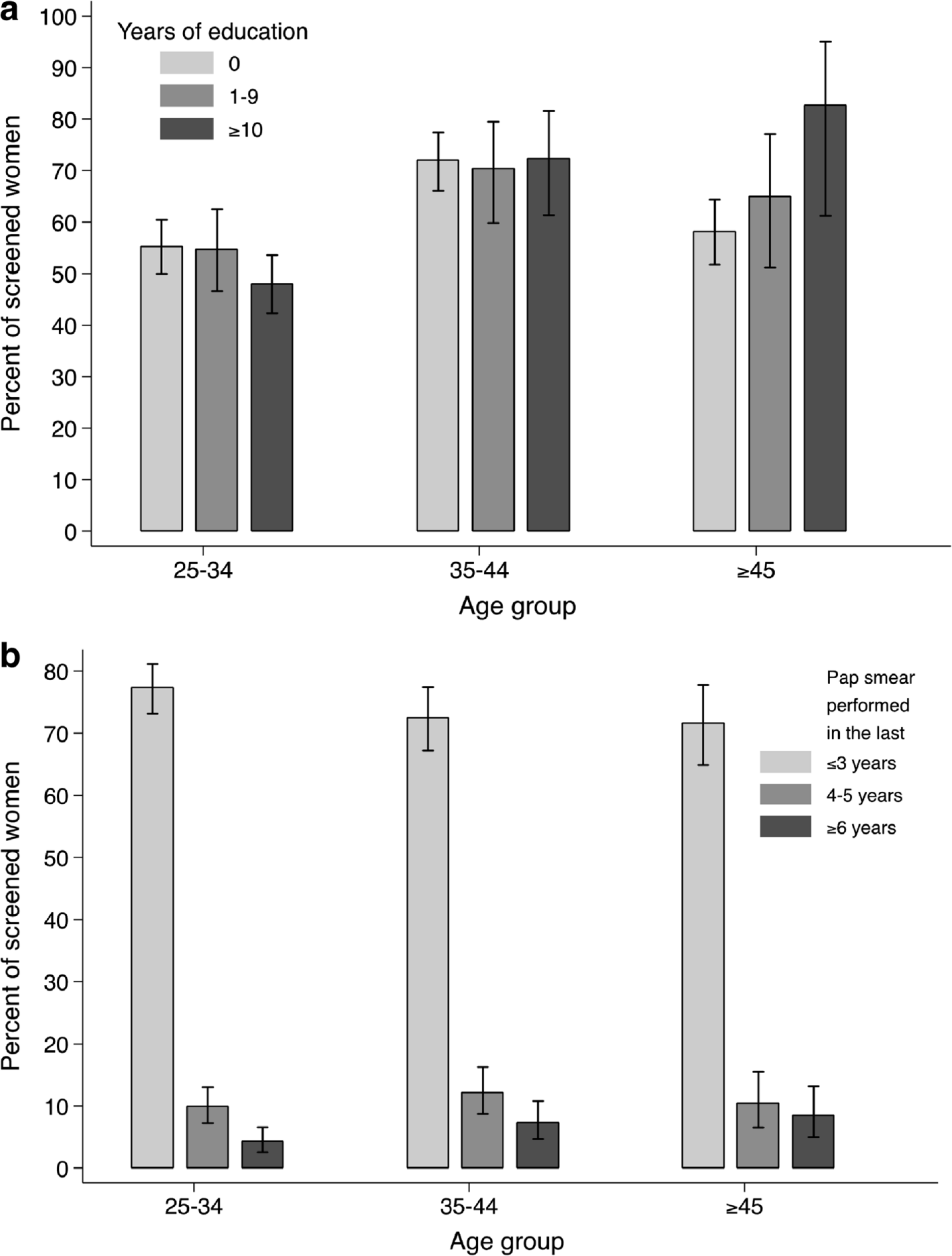
**Percentage of screened women by age group and years of education, 1,620 women (a); or years elapsed since last Pap smear, 959 previously screened women (b); Thimphu and Lungthenphu, Bhutan.**

Education level was positively and significantly associated with previous screening attendance among women age 45 or above (*χ*^2^ for trend = 5.3 p = 0.02) but not in younger age groups (p-value for heterogeneity = 0.06) (Figure 2a). Among 959 previously screened women, over 70% in every age group reported a Pap smear within the last three years and few reported an interval of six years or more (Figure 2b).

Table 2 shows screening history among 1,503 women from Thimphu according to the place in which they were contacted and whether they accepted the invitation. Among women invited from home, the invitation compliance was 31%. It was 28%, 36%, and 31% in women <35; 35-44, and ≥45, respectively (data not shown). The largest fraction of lack of screening (75%) was found among women who were contacted at home but did not participate in our study (OR for lack of screening *vs* women who were contacted at the clinic = 4.3; 95% CI: 3.4-5.6). We notice that only 105 (21.9%) of 479 never-screened women who were invited from home could eventually be screened for the first time in the framework of our study. Past screening attendance in all women contacted at home was 33.9% (95% CI: 30.5-37.3) and we considered it a proxy of population-based coverage (Table 2).

**Table 2:** **Odds ratio and 95% confidence interval for lack of prior screening by place invited and study participation, 1,503 women, Thimphu, Bhutan**

**Place of invitation**	**Total**	**Ever screened N. (%)**	**Never screened N. (%)**	**Odds ratio** ^**a**^	**95% confidence interval**
Clinic	778	457 (58.7)	321 (41.3)	Ref. cat	
Home, participants	225	120 (53.3)	105 (46.7)	1.3	1.0 - 1.7
Home, non-participants	500	126 (25.2)	374 (74.8)	4.3	3.4 - 5.6
Home, all	725	246 (33.9)	479 (66.1)	2.9	2.3 - 3.6

^a^Adjusted for age.

**Figure 3:**
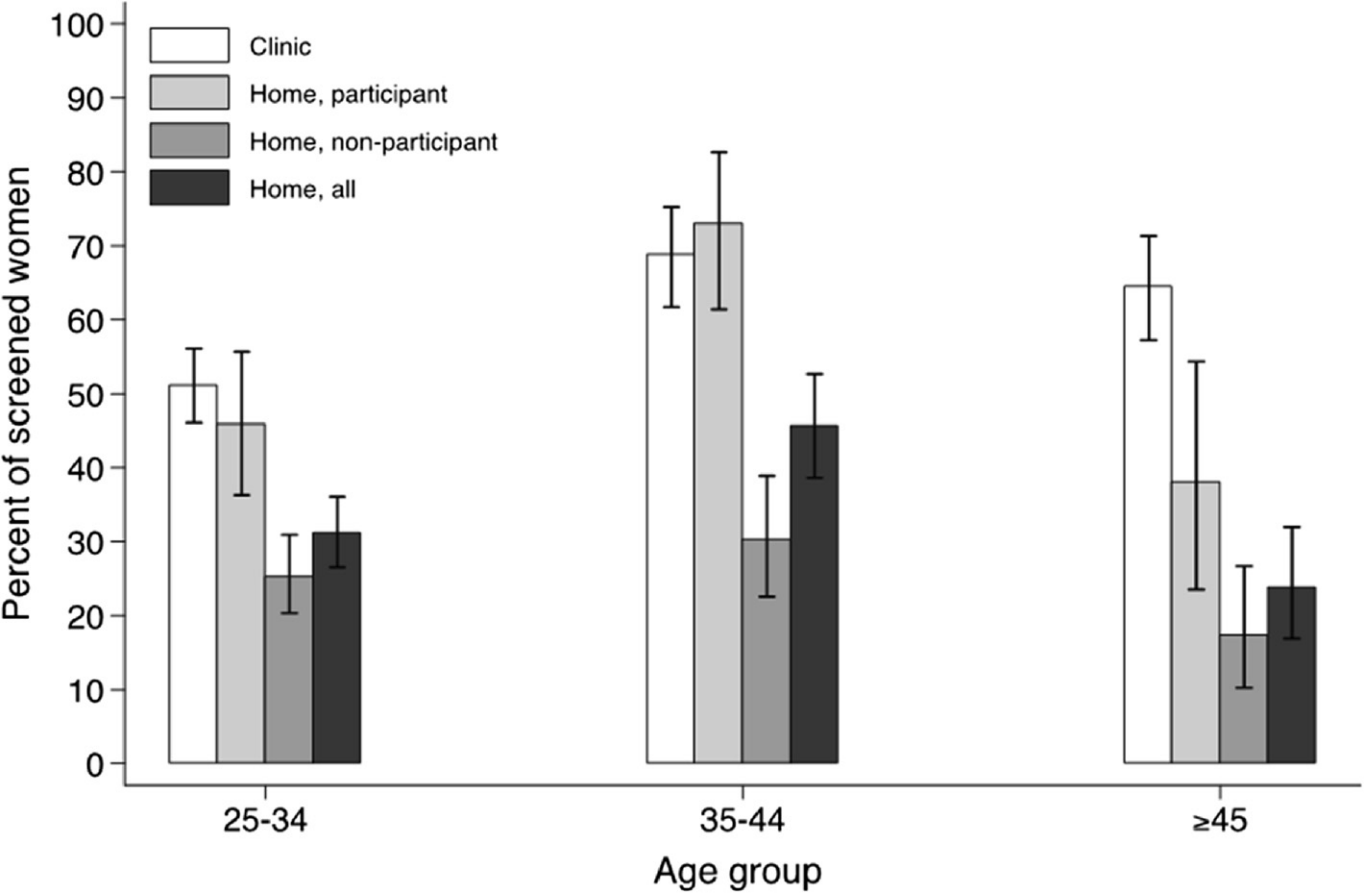
**Percentage of screened women by place contacted, study participation, and age group, 1,503 women, Thimphu, Bhutan.**

Figure 3 shows that the gap in coverage by place in which women were invited and according to study participation increased with age. Among women age 45 years or older screening coverage among all women who were contacted at home was 23.9% (95% CI: 16.9-32.0).

## Discussion

Our study showed for the first time that the cervical cancer screening program in Thimphu reaches approximately one third of women age 25 or older and lack of previous screening was especially frequent among women who refused the invitation to join our study. They, therefore, missed the opportunity to be screened for the first time in the framework of our study.

**Figure 4:**
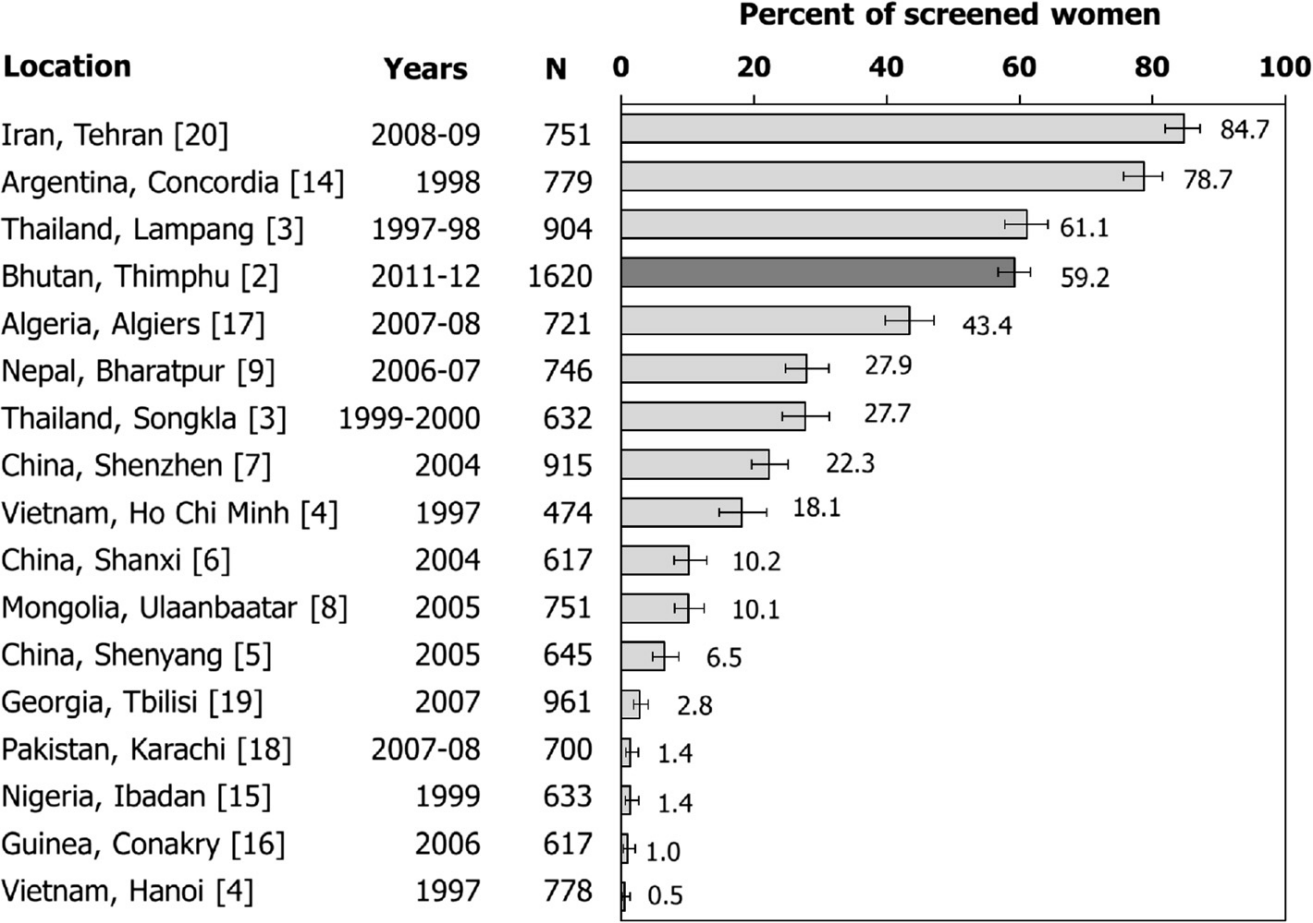
**Percentage of women age ≥25 years who reported previous cervical cancer screening in low/middle-income countries.** IARC HPV surveys, 1997-2012.

Among women who took part in our study, previous screening attendance was relatively high (59%) and better than in the majority of the 16 low/middle-income populations who were formerly studied in methodologically consistent IARC HPV Surveys, i.e., similar recruitment protocol, age distribution, participation level, and location in places in which at least minimum cervical screening expertise and infrastructure were already present [2-9,14-20] (Figure 4). Much lower levels of screening attendance were found in Nepal, China, Pakistan, and Vietnam. Only in Tehran, Iran; Argentina, Concordia; and Lampang, Thailand was the proportion of previously screened women larger than in Bhutan. We notice that the proportions of ever-screened women in previous IARC HPV Surveys were probably affected by a similar participation bias as in the figure from Bhutan.

Among study participants in Thimphu and Lungthenphu, screening history was also significantly affected by several factors. Past screening attendance was more frequently reported by women 35 to 45 years old than by younger or older women. Being married, having had children, and using any type of contraceptive method were also significantly and positively associated with past screening attendance. Our findings provide, therefore, additional evidence [21,22] on the limitations, in terms of population-based coverage, of mainly linking screening offer with attendance at perinatal care and family planning clinics.

Manual workers, farmers, shop-keepers and saleswomen reported previous screening less often than housewives but this was not the case for clerical staff, teachers and health workers. Economic reasons are unlikely to explain lack of screening in Bhutanese women because of the universal access to free health care. However, inconvenient opening hours at screening clinics and earning losses from attending may well discourage some women from being screened. Interestingly, education level was associated with screening attendance only among women aged 45 years or above. A larger fraction of never-screened women was also found in the Lhotsampa minority, a predominantly Hindu ethnic group in a predominantly Buddhist country. Finally, lack of previous screening was more frequent among women at higher cervical cancer risk, i.e., the few who reported two sexual partners or more and the many who were HPV-positive.

Cervical cancer screening is still not widely understood and accepted by Bhutanese women. A recent report [10] has explored knowledge on and attendance of cervical cancer screening in university graduated, and mainly unmarried, women (mean age 23 years). The study revealed that, despite national recommendations to start at age 20, approximately 95% of Bhutanese female university graduates had never been screened. The most frequently reported reasons for not attending screening were a) no awareness of the need for a Pap smear (57%), b) embarrassment of being examined by male health professionals (24%), and c) fear of being diagnosed with cancer (20%). Similar reasons may explain lack of screening in a fraction of women in our present study.

Like in most low/middle-income countries and in all areas included in Figure 4, the population in the capital of Bhutan is rapidly growing, census is incomplete, and addresses inaccurate. The national screening program does not include an individual active call-and-recall system and women are invited to join the screening program through a variety of ways, e.g., mobilization at the community level, leaflets, posters, and messages on media. An accurate estimation of population-based screening coverage was, therefore, lacking. We have clearly shown that past screening attendance among women who accepted to join our study was affected by participation bias. Thanks to the information on screening history that was obtained during home visits we have been at least partially able to overcome this bias. Past screening attendance was 59% among all participants but 34% in women contacted at home, and only 25% in women contacted at home who refused to participate in our study. Low screening attendance in women age 45 or older is especially concerning as it points to a large pool of undetected severe cancerous and precancerous cervical lesions in Thimphu [23]. Also of concern, less than one third of women invited from home joined our study.

Strengths of the present study include the large number and broad age range of participants, and the combination of different sampling methods that gave us the possibility to tackle participation bias. Some control of self-reported screening history could be made among women who attended study clinics by crosschecking women’s answers with the records of JDWNRH, Department of Laboratory Services, the only cytology provider in Thimphu. Screening history collected at home among non-participants may have been less accurate than information obtained during study interview for participants, as some women may not have fully understood the question. Obviously, we have no information on screening attendance among women who were never found at home by social workers, despite repeated attempts.

## Conclusions

The transition from an opportunistic screening to an all-reaching population-based screening is yet to be achieved in the capital of Bhutan, let alone in the entire country. An active individual call and recall system has been a key factor in improving screening coverage in western countries [22] but invitation at home did not work very well in never-screened women in Thimphu.

A wider offer of screening also outside maternal and child health clinics, and better health education are needed. *Ad hoc* methods to target never-screened women, e.g., through campaigns set in working places, markets, religious events, and other public meeting places, are however the priority in Bhutan. As shown in the Netherlands [24], self-collection of vaginal samples in combination with HPV testing may overcome women’s reluctance to attend a gynaecological examination in Thimphu and also facilitate the performance of cervical cancer screening in rural and high-altitude areas in Bhutan.

## Abbreviations

CI: Confidence interval
HPV: Human papillomavirus
IARC: International Agency for Research on Cancer
ICC: Invasive cervical cancer
JDWNRH: Jigme Dorji Wangchuck National Referral Hospital
OR: Odds ratio
